# Cytological changes in the oral mucosa after use of a mouth rinse with alcohol: A prospective double blind control study

**DOI:** 10.4317/medoral.18843

**Published:** 2012-10-20

**Authors:** Jose V. Bagan, Francisco Vera-Sempere, Cristina Marzal, Ana Pellín-Carcelén, Ezequiel Martí-Bonmatí, Leticia Bagan

**Affiliations:** 1 Professor of Oral Medicine. Valencia University. Chairman Service of Stomatology and Maxillofacial Surgery, University General Hospital, Valencia; 2 Professor of Pathology. Valencia University. Chairman Service of Pathology, University La Fe Hospital, Valencia; 3 Dentist. Assistant professor of Oral Medicine. Valencia University; 4 Biologist. Service of Pathology, University La Fe Hospital, Valencia; 5 Pharmacologist. Service of Pharmacology, University General Hospital, Valencia; 6 Collaborator in Oral Medicine, Valencia University, Spain

## Abstract

Aim: The aim of this preliminary study was to detect cytological changes in the oral mucosa after using a mouth wash with alcohol. Material and Methods: A prospective double-blind, controlled study was performed, for 6 months. Group 1 consisted of 30 subjects who used a mouth rinse with 26.9% of alcohol [Listerine®] and Group 2 consisted of 30 subjects who used a mouth rinse with the same ingredients but with no alcohol. We obtained three cytological samples from the oral mucosa. The presence of cytological atypia, binucleation and karyorrhesis, and type of cells were studied. We also used a fluorescent in situ hybridization technique (FISH) in 15 samples in each group, for the micronucleus. Results: We found no clinical mucosal alteration after using the mouth wash at the end of the study in either group. We observed no cytological differences between the groups at the end of the study (p>0.05). Regarding the study of the micronucleus by FISH, we observed no significant difference between the groups (p>0.05). Conclusions: Our results showed no cytological alteration in patients using a mouth rinse with alcohol, but these findings should be considered preliminary results, to be confirmed in a greater sample of patients.

** Key words:**Mouth wash, oral mucosa, cytological change, alcohol.

## Introduction

Mouth washes are used widely in dentistry. They usually contain water with some active components, such as antiseptics, antibiotics, antifungal, astringents, and anti-inflammatory substances (1). In addition to the mechanical removal of dental plaque, some mouth washes have been described as enhancing the removal process and elimination of bacteria (2).

Two antiseptic mouth washes have been approved by the American Dental Association (ADA), based on clinical trials: Peridex (Zila Pharmaceuticals, Phoenix, AZ, USA) is a 0.12% solution of chlorhexidine and Listerine® (LN; Pfizer Consumer Healthcare, Morris Plains, NJ, USA; essential oil, AE). The active ingredients of Listerine® are eucalyptol 0.092%, menthol 0.042%, methyl salicylate 0.060%, and thymol 0.064% for anti-plaque/anti-gingivitis. The inactive ingredients are water, alcohol (26.9%), benzoic acid, poloxamer 407, sodium benzoate, and caramel. The ADA stated that “The Council on Scientific Affairs’ acceptance of Listerine® Antiseptic is based on findings (3-5) that the product is effective in helping to prevent and reduce gingivitis and plaque above the gumline, when used as directed.”

Ethanol is used as a solvent for the active agents in many commercially available mouth rinses, with concentrations ranging from 6% to 26.9% (6-9). However, Kowitz et al. (10) described some adverse effects after using these mouth washes, such as epithelium desquamation, ulcerations, gingivitis, and petechiae. (11) also presented two cases with white plaques associated with the use of Listerine®.

Some authors have stated that oral cancer is increased or contributed to by the use of alcohol-containing mouth rinses (12). Guha et al. (13) described that daily mouth wash use may be an independent cause of cancers of the head, neck, and esophagus.

In contrast, other authors found no evidence to support any relationship between mouth washes and oral cancer (14,15). Addi-tionally, Elmore & Horwithz (16) noted that neither the data for the overall association nor an analysis of patients without other clinical risk factors supported a link between mouth wash use and oral cancer.

Cytological studies have been used to analyze possible oral mucosal changes after using mouth rinses with alcohol (17). Thus, based on these reported discrepancies, we sought to analyze, in a preliminary prospective case-control study, possible cytological changes using a combined analysis of the micronucleus (MN) and FISH technique in patients using a mouth wash containing alcohol.

## Material and Methods

This study was conducted by the Oral Medicine Department at Valencia University, and in the Pathology Department at La Fe University General Hospital, Valencia, Spain, in the period from 2009 to 2010. All patients provided written informed consent and the research was approval by the Ethical Committee at Valencia University.

This was a double-blind, prospective, randomized clinical trial that took place over 6 months. There were 60 patients; the mean age was 41.27±6.26. There were 19 (31.7%) males and 41 (68.3%) females.

Inclusion criteria were patients who attended the Faculty of Dentistry to be examined for general dental problems with their teeth. They were between 30 and 50 years old and voluntarily accepted to use the provided mouth wash daily for 6 months. Exclusion criteria were smokers and ex-smokers who quit in the last 5 years, daily drinkers of more than 80 mL alcohol/day, pregnant women, those taking xerostomising drugs, and use of mouth rinses in the 2 months prior to the study.

We randomly assigned, in a double-blind manner, one of the two mouthwashes to the 60 subjects. Finally, 30 subjects used a mouth wash with 26.9% of alcohol [Listerine®] (group 1) and 30 subjects used another mouth rinse with the same components but with no alcohol (group 2).

Methods

A dental exam was performed in every subject at the baseline. We analyzed the DMF index (18), plaque index (19), bleeding index (20), and the average periodontal depth and periodontal loss of insertion. The authors of this article, trained in oral medicine, also examined the oral mucosa. There was no statistically significant difference between the groups at baseline in dental findings, age, or gender ( Table 1).

**Table 1:**
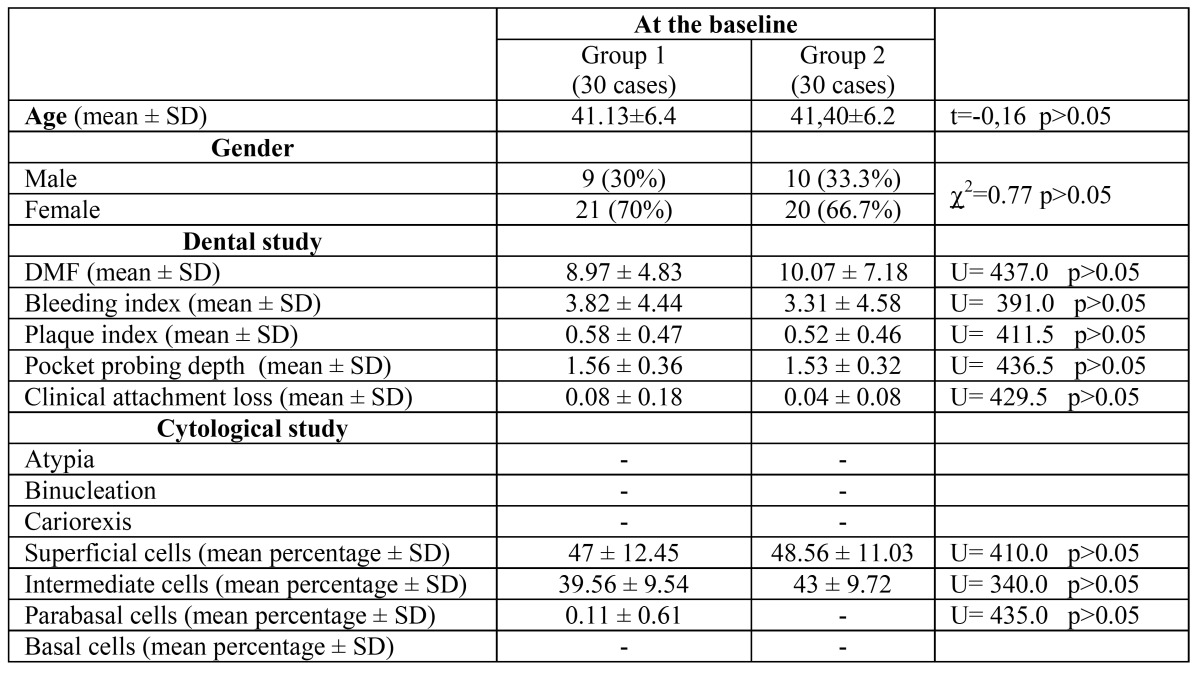
Comparison of dental status and cytological findings at baseline between groups.

In each subject, we took three cytological smears, before starting with the mouth rinse and after 6 months. Two of the three cytological samples were taken by scraping from the lateral border of the tongue and the buccal mucosa. The other sample was obtained after rinsing the mouth with 5 mL of sterile physiological saline, which was then collected for analysis.

Cytological samples were processed as follows: after washing with physiological saline solution, the resulting cellular material (3-5 mL) was placed in a sterile tube and centrifuged (10 min, 1500 rpm). The supernatant was discarded and a smear preparation of the sediment was mounted on a slide, followed immediately by fixation in 95% ethanol through repeated immersion for 15 s. The specimen was then subjected to Papanicolaou staining (Harris hematoxylin, EA50, Orange G, eosin). The entire cytological study was conducted by the same pathologist (Prof. F Vera), evaluating the following parameters:

- Proportion of superficial, intermediate, parabasal, and basal Malpighian cells in the smear.

- Presence of nuclear atypia.

- Presence of binucleation and karyorrhexis.

Additionally, a random sample was taken in 10 patients (5 from the control group and 5 from the study group) at the end of the study, after using the mouth rinses, for cytological analysis. The thin layer (ThinPrep) metho-dology was used, followed by fluorescent in situ hybridization (FISH) (21). In each of these 10 patients, and in the three samples obtained (tongue, cheek mucosa, oral wash/rinse), conventional cytological observation with Papanicolaou staining was used to evaluate the presence of micronuclei (MN). These were defined as the presence of smaller diameter, perinuclear chromatinic bodies (22). In total, 100 well-preserved cobblestone cells (intermediate or superficial) were counted per sample, avoiding zones with abundant flora.

The FISH technique was used for 10 cells in each of the cytological specimens. Accordingly, we analyzed a total of 150 cells with micronuclei in each group.

Samples corresponding to these three locations were processed with ThinPrep 5000 (Hologic). Briefly, the samples were subjected to a first centrifugation step (2800 rpm, 5 min); the supernatant was discarded and the pellet was subjected to a second centrifugation step and washing (5 min, 2800 rpm) with Cytolyt (Hologic) solution. The pellet was then aspirated and placed in a vial with PreservCyt (Hologic) solution for 15 min. The sample was finally subjected to ThinPrep 5000 processing for 2-3 min, followed by slide preparation for Papanicolaou staining after cytological fixation in 96% alcohol for 15 min.

Papanicolaou staining was carried out using a Leica automated staining system. Likewise, in a consecutive step, a second slide preparation was obtained and independently processed for micronuclear analysis with the FISH technique. The slide for FISH analysis was fixed in methanol-acetic acid solution (3:1) for 20 min at room temperature. After digestion with pepsin (37°C, 3-10 min), washing was conducted with 2× SSC buffer, with dehydration in a rising alcohol gradient. FISH was then performed using the All Human Centromere Probe, Green (Kreatech Diagnostics, Amsterdam, The Netherlands). Hybridization was carried out in two steps: the first at 80°C for 5 min, and the second at 37°C for 16 h, using a DakoCytomation hybridizer with a wet chamber. After hybridization, the corresponding washing steps were carried out, followed by mounting with DAPI/Fade (Master Diagnostica). The mounted slides were kept in a dark chamber at 4°C until the time of microscopic examination, when the results were visualized using a Nikon Eclipse 80i epifluorescent microscope to obtain ×1000 micrographs with a refrigerated high-resolution Nikon digital camera for FISH analysis.

Statistical analysis 

We used Student’s t-test for comparison of continuous and quantitative values between groups if the samples had a homogeneous distribution; otherwise, we used the Mann-Whitney U-test. A Wilcoxon test was used to contrast the homogeneity of percentages in both groups. A Pearson’s X2 test was performed to compare the association or independence between qualitative values. Finally, the proportion of changes in the variables was analyzed using the McNemar test in related samples. We considered differences to be statistically significant if p<0.05.

## Results 

Regarding cytological findings, we found no statistical difference between the groups at baseline, before starting the mouth washes ( Table 1; p>0.05). We also found no clinical mucosal alteration after using the mouth wash at the end of the study in either group.

When analyzing the cytological differences between both groups at the end of the study (6 months of using mouth washes) there was no statistically significant difference ( Table 2). We found no case of atypia in either group. We detected one case with binu-cleation in group 1 (3.3%) but none in group 2. This case with binucleation was found both in the buccal mucosa scrapings and in the rinse sample, but no alteration was found in the tongue scrape. We found one case of karyorrhexis, but only in the control group.

**Table 2:**
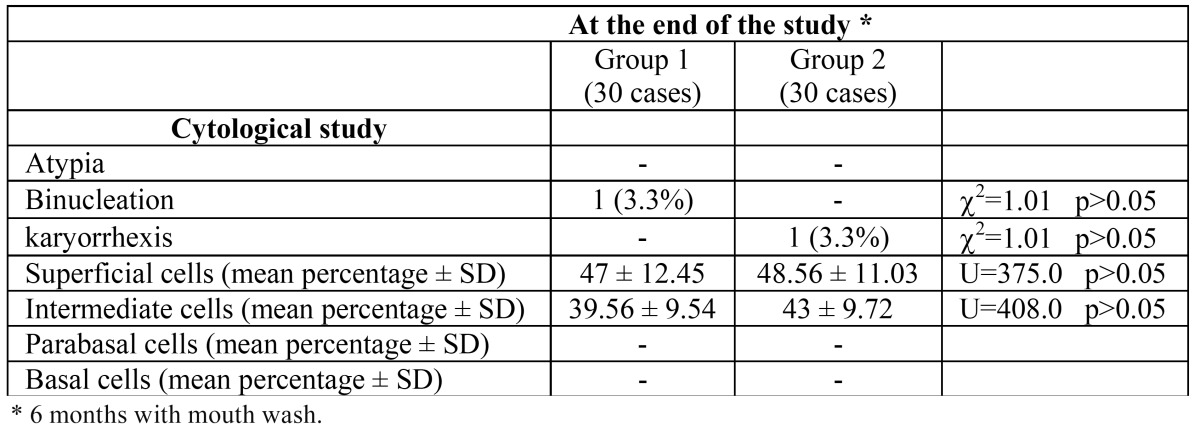
Comparison of the cytological findings at the end of the study between groups.

There was no statistically significant difference in the percentage of superficial, intermediate, parabasal, or basal cells between the groups ( Table 2; p > 0.05).

**Figure 1:**
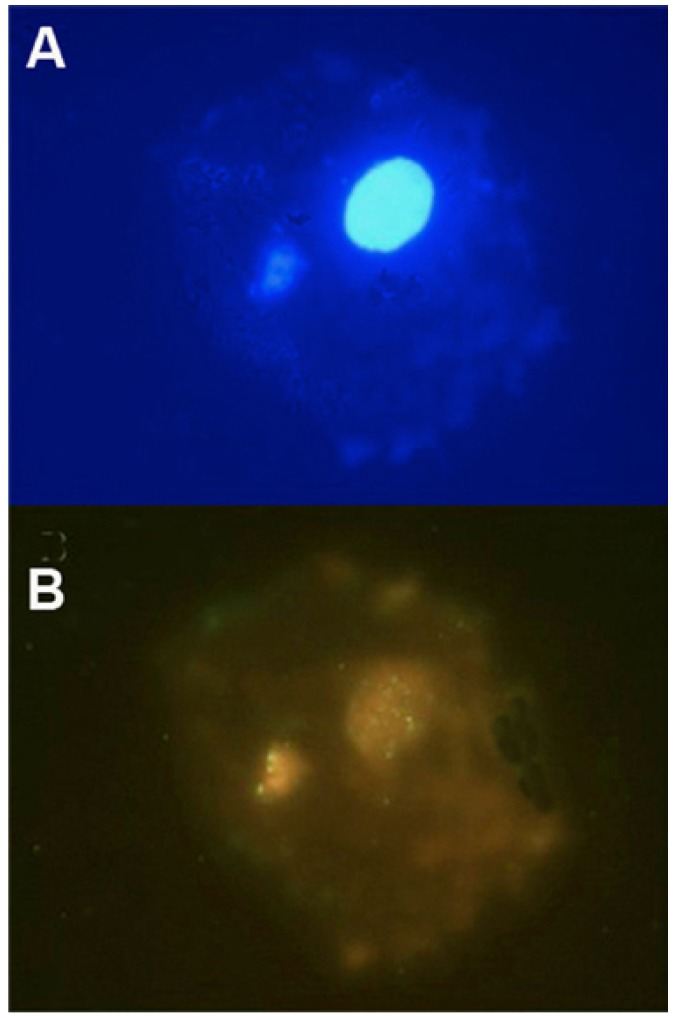
Micronucleus in oral squamous cell observed by FISH technique: A) DAPI stain, B) FISH using the “All Human Centromere Probe” revealed green centromeric signals in both the nucleus and micronucleus (FISH, 1000×).

We studied five cases for micronucleus by FISH analysis in both groups. We found nine cases with MN in group 1 in a sample of 100 cells; 86% of these positive cells showed positivity by the pancentromeric probe used (Fig. 1). We detected seven cases with MN in the control group; 83% were positive for the pancentromeric probe. These results showed no significant difference between the groups (p>0.05).

## Discussion

Mouth rinses are used widely worldwide, mainly for their capacity to control dental plaque and gingivitis. Daily use of mouth rinses has been recommended for the prevention and control of caries and periodontal disease (23,24). According to Silverman & Wilder (2), when used in conjunction with brushing and flossing, they are an important method for reducing plaque, gingivitis, and preventing or minimizing periodontal disease. Mouth rinses have also been reported to be effective in the management of radiated head and neck cancer patients (25-27).

Many mouth rinses with antiplaque properties contain pharmaceutical-grade denatured alcohol as a vehicle. Concern has been raised regarding the potential for alcohol-containing rinses to cause adverse effects. In fact, the use of alcohol-containing mouth rinses should be restricted in high-risk populations according to (7). Periodontal disease, as indicated by poor condition of the mouth and missing teeth, and daily mouth wash use may even be independent causes of head, neck, and esophagus cancers (28).

Further, according to McCullough & Farah (12), there is sufficient evidence to accept the proposition that alcohol-containing mouth washes contribute to an increased risk of developing oral cancer. In contrast, other authors found no evidence to support any risk between mouth rinses and alcohol. Cole et a. (15) identified nine English-language epidemiological studies that made reference to mouth washes. They concluded that it was unlikely that the use of mouth washes that contain alcohol increased the risk of developing cancer. Lemos & Villoria (29) stated that the correlation between oral cancer and alcohol-based mouth rinses was so small, weak, inconsistent, and even contradictory that any kind of risk warning to patients would be uncalled for. The role of alcohol in oral tissues has also been studied and non-cytotoxicity and the absence of histopathological effects were found by Koschier et al. (30).

In an excellent epidemiological study by La Vecchia et al. (14), the link between mouth wash use, specifically, alcohol-containing mouth wash, and oral cancers, was not supported by epidemiological evidence. Finally, according to Silverman & Wilder (2), antimicrobial mouth rinses are safe and effective.

We performed a prospective double-blind controlled study to analyze possible alterations in oral mucosal cells after using a mouth wash with alcohol for a period of 6 months, and compared it with another without alcohol. Other authors, such as Carlin et al. (17) only exposed their patients to mouth rinses with alcohol for 2 weeks. This is one of the most significant differences between their study and ours.

Another issue is the strict inclusion criteria we used. We only admitted cases between 30 and 50 years who were not smokers or heavy drinkers. We excluded those using xerostomic drugs and patients who had not used mouth rinses within 2 months prior to the study. Another significant feature was that there was no difference between the dental conditions at baseline between the groups. This made our groups highly comparable and homogeneous before exposure to the mouth washes.

After 6 months we found no clinical alteration in the oral mucosa in either group, in contrast to the findings of Kowitz et al. (10) and Bernstein (11). Dórea et al. (31), studied nuclear alterations suggestive of apoptosis: karyorrhexis, condensed chromatin, and pyknosis. The protocol they used was also used by others (22,32,33). They found 393 karyorrhesis, 803 condensed chromatin, and 136 pyknosis in 51,153 cells analyzed in apparently normal mucosa.

We found no case of atypia after 6 months using the mouth rinse in either group. We only detected one case of binucleation in the Listerine® group, but none in the control group. Regarding karyorrhexis, we found only one case, but in the control group (p>0.05). The percentage of superficial, intermediate, and parabasal cells showed no statistically significant difference between the groups.

DNA damage is a crucial event in carcinogenesis (34). The study of DNA damage in exfoliated buccal cells is a minimally invasive method for monitoring populations for exposure to genotoxic agents (35). The presence of micronuclei and other nuclear anomalies within these cells has been shown to be a useful tool with regard to DNA alterations. The International Human Micronucleus (HUMN) Project (www.humn.org) was founded in 1997 to coordinate worldwide research efforts aimed at using micronucleus assays to study DNA damage in human populations. The MN assay in exfoliated buccal cells is a minimally invasive and potentially useful method for monitoring genetic damage in humans. Recommendations for MN studies have been proposed (36).

The comet assay is considered a quick and reliable method of analyzing DNA damage in a single cell. It is also described as being highly sensitive for detecting genotoxicity (37). The potential of these two methods, the comet assay and MN, can be enhanced by combination with fluorescence in situ hybridization (FISH) techniques. FISH is used in genetic toxicology for the analysis of chromosome damage (21,38-40). Thus, FISH is recognized as a valuable addition to MN assays (41).

Thus, we thought that the combination of MN and FISH techniques would be more appropriate to analyze possible alterations in the oral mucosa after the use of mouth rinses containing alcohol. As a result of this preliminary study, we found no statistically significant difference between the groups.

This study provides support for continuing to use this method, combining MN and FISH, to detect cytological alterations in these patients, although we recognize that a larger number of cases should be analyzed after this preliminary analysis to establish more conclusive results. Meanwhile, the recommendation of Ready et al. (7) that the use of alcohol-containing mouth-rinses should be restricted in high-risk populations should be kept in mind.
